# Controlled Somatic and Germline Copy Number Variation in the Mouse Model

**DOI:** 10.2174/138920210793176038

**Published:** 2010-09

**Authors:** Yann Hérault, Arnaud Duchon, Damien Maréchal, Matthieu Raveau, Patricia L. Pereira, Emilie Dalloneau, Véronique Brault

**Affiliations:** 1 Institut de Génétique et de Biologie Moléculaire et Cellulaire (IGBMC), INSERM U964, CNRS UMR7104, Université de Strasbourg, Illkirch, France; 2 Institut Clinique de la Souris (ICS), INSERM U964, CNRS UMR7104, Université de Strasbourg, Illkirch, France

**Keywords:** Chromosomal engineering, Cre/loxP, mosaic genetics, duplication, deletion.

## Abstract

Changes in the number of chromosomes, but also variations in the copy number of chromosomal regions have been described in various pathological conditions, such as cancer and aneuploidy, but also in normal physiological condition. Our classical view of DNA replication and mitotic preservation of the chromosomal integrity is now challenged as new technologies allow us to observe such mosaic somatic changes in copy number affecting regions of chromosomes with various sizes. In order to go further in the understanding of copy number influence in normal condition we could take advantage of the novel strategy called Targeted Asymmetric Sister Chromatin Event of Recombination (TASCER) to induce recombination during the G2 phase so that we can generate deletions and duplications of regions of interest prior to mitosis. Using this approach in the mouse we could address the effects of copy number variation and segmental aneuploidy in daughter cells and allow us to explore somatic mosaics for large region of interest in the mouse.

## INTRODUCTION

Our knowledge about human genetic variation has considerably evolved in the past few years with the development of genome-wide technologies unrevealing structural variations such as oligonucleotide microarray technologies and the next-generation sequencing with “paired-end” methods [1] that enable to screen the genome at a submicroscopic level. The identification of frequent copy number variants (CNVs) between individuals, including deletions and duplications of DNA sequences has definitely changed our view of genome integrity. Thousands of different CNVs have been identified (http://projects.tcag.ca/variation) overlapping a large number of genes [2, 3] and accounting for about 13% of the human genome. Estimates suggest that CNVs account for up to 24 Mb [3, 4] of the genetic difference between individuals, exceeding single nucleotide polymorphism (SNP) (2.5 Mb) [5]. 

In addition of being a major source of genetic diversity in the human population, CNVs are also responsible for various pathological conditions such as cancer, aneuploidies and contiguous gene syndromes (CGS). The first CNVs that were recognized were due to large chromosomal rearrangements that were visible under light microscope, causing well known genomic disorders such as Down (trisomy 21), Turner (monosomy X), Fragile X (Xq27.3) or Cri du Chat (del 5p-) syndromes [6]. These genomic disorders are often sporadic as they are de novo mutations leading to severe developmental deficits not compatible with the individual having offsprings. However, some CNVs can be inherited in a Mendelian way: Charcot-Marie-Tooth disease type 1A is a dominant neuropathy associated to a duplication of the gene for peripheral myelin protein 22 (PMP22) [7], whereas a deletion of the b-globin gene cluster is responsible for the recessive anemia-thalassemia [8]. The number of recognized genomic disorder has been drastically increasing since the 1980s by the constant discovery of submicroscopic (<5 Mb) genomic deletions and duplications associated with pathological phenotypes [9, 10]. Microdeletion 15q11.2q12 was associated with Prader-Willi syndrome in 1981 [11], a 17p13.3 deletion was found in Miller-Dieker lissencephaly syndrome [12], a 1.5 to 3.0-Mb hemizygous deletion of chromosome 22q11.2 causing haploinsufficiency of the *TBX1* gene was discovered in DiGeorge syndrome [13], whereas the first recurrent microduplication was identified in 1991 in patients with Charcot-Marie-Tooth disease type 1A (CMT1A) [7]. In particular, the discovery of new CNVs has increased the diagnostic of mental retardation symptoms that affect 2-3% of the population as define new recognized syndromes by 15-20%. Moreover, CNVs are increasingly associated with complex traits such as autism [14, 15], schizophrenia [16] or epilepsy [17, 18] and susceptibility to infectious diseases such as HIV [19], Crohn disease [20, 21], psoriasis [22] or malaria [23]. 

A major mechanism by which rearrangements can cause a phenotype is by altering the copy number of a gene (or genes) sensitive to a dosage effect and influencing its expression [24]. But deletions and duplications can also cause gene expression perturbation through positional effect [25]. This position effect can be either the result of the physical dissociation between the transcription unit and its regulatory sequences that can sometimes lie as far as 1 Mb away from it [26, 27], or the result of the alteration of local or global regulation of chromatin structure [28-31]. These effects have been documented by some human disease states. For example, Aniridia (absence of the iris) due to haloinsufficiency of *PAX6* at 11q13 has been shown to result from a chromosomal rearrangement that disrupts the region downstream of PAX6 transcription unit [32-35]. Human diseases resulting from aberrant gene expression through altered chromatin structures include FSHD (facioscapulohumeral dystrophy) (MIM 158900), a neuromuscular disorder affecting the facial and shoulder girdle muscles and one case of α-Thalassemia (MIM 141800) in which the *HBA2* gene has been silenced by methylation of its promoter region by an antisense RNA derived from the truncated neighboring *LUC7L* gene on the opposite strand [31]. Genomic rearrangements of the genome can also trigger phenotypes by unmasking of recessive mutations or functional polymorphism of the remaining allele in the case of deletions. Hence, it was shown that, in Sotos syndrome that is due to del 5q35, the severity of the disease depends on the polymorphism of the remaining copy of the coagulation factor 12 gene [36]. 

CNVs often arise from meiotic recombination between highly similar (<97% sequence identity) duplicated sequences (termed low-copy repeats or LCRs) located less than 10 Mb apart. LCRs represent up to 5% of the haploid human genome [37] and can cause genomic instability as they can be at the origin of misalignment of chromosomes or chromatids and cause nonallelic homologous recombination (NAHR), resulting in unequal crossing-over [38]. But other mechanisms have been also proposed to be responsible for CNVs formation: nonhomologous end joining (NHEJ) is a mechanism that repairs DNA double strand breaks and, when defective, can lead to translocations and telomere fusion, hallmark of tumor cells [39]. Fork stalling and template switching (FoSTeS), a DNA replication error mechanism, has recently been shown to play an important role in the origin of nonrecurrent rearrangements with a complex structure leading to genomic disorders of various size [40-42]. Several studies have predicted that NHEJ or FoSTeS are at the origin of some CNVs observed in some cases of Duchenne muscular dystrophy, Smith-Magenis syndrome and Pelizaeus-Merzbacher disease [43, 44]. These mechanisms have the particularity of leading to CNV mosaicism. Occurrence of somatic mosaicism has been reported for chromosomal aberrations connected with diseases and leading to a milder phenotype of the disease [45, 46], arising somatically in cancers [47-49], or in the case the rearrangements of the immunoglobulin and T-cell-receptor genes [50]. In healthy individuals, more and more data have recently accumulated showing inter- and intra- tissues mosaicism for entire chromosome aneuploidies in germ cells, placenta, brain, skin, liver and blood [51, 52], although their occurrence remains unclear, due to the techniques that use pooling of large number of cells to study CNVs. Recent studies have started to point out at the importance of CNVs somatic mosaicism: extensive chromosomal instability and de novo recurrent CNVs have been reported in human cleavage-stage [53] and in mouse embryonic stem cell [54] respectively and somatic CNV mosaicism has been reported in different human tissues and organs [55] with studies on monozygotic twins revealing putative de novo somatic CNV events [56]. In a paper published this year, the group of Anja Weise at the Institute of Human Genetics in Magdeburg (Germany) provided the first evidence of somatic mosaicism for CNV between different cell types of one individual [57]. They used an approach that was defined to determine chromosomal parental origin in single cells based on fluorescent in situ hybridization (‘parental-origin-determination fluorescence in situ hybridization’ or pod-FISH) [58] to visualize CNVs on homologous chromosomes metaphase spreads from 10 healthy individuals and found CNV variation between different cell types but not within one cell type. They hence propose that there is early embryonic chromosome instability which results in stable mosaic pattern in human tissues.

While CNV is now recognized as a major source of genetic variability between individuals, their biological consequences are largely under investigated. Analysis of their pathological role and molecular mechanism are hard to investigate in human and require an animal model, genetically, morphologically and physiologically closer. The mouse constitutes a model organism of choice with an anatomy, physiology and genetics highly similar to humans. 80% of mouse genes have an orthologous counterpart in the human genome [59] and 99% have a sequence match. Homologous genes are found in the same order and relative orientation in large blocks of conserved syntenic regions. In addition, the ability to manipulate the mouse genome has made the mouse the primary mammalian genetic model organism. CNV can be artificially created using various chromosome engineering techniques that enable to precisely manipulate large genomic regions. Especially, the technology based on the Cre-*lox*P system is used to generate new chromosomes carrying deletions, duplications, inversions and translocations in targeted regions of interest and to study contiguous gene syndromes as well as normal developmental processes associated with CNV. 

## I. GERM LINE GENETIC ENGINEERING USING EMBRYONIC STEM (ES) CELLS

Modelling chromosomal rearrangements such as deletions, duplications, inversions or translocations in the mouse genome has been made possible by combining gene targeting in embryonic stem (ES) cells [60-63] with site-specific recombinase (SSR) systems such as the Cre-*lox*P [64]. Gene targeting enables to introduce small DNA sequences known as *lox*P sites to predefined loci by homologous recombination. Subsequent expression of the bacteriophage Cre recombinase that catalyses recombination between *lox*P sites [65-68] and can act in the mammalian genome without any cofactor, allow to generate large chromosomal rearrangements either in ES cells and then transmitted to the mouse by injecting the transformed ES cells to blastocysts, or directly in the mouse. The size of the *lox*P, small enough to be introduced very easily by genetic engineering and large enough to avoid problems associated with cryptic occurrence in eukaryotic genomes, has made the Cre-*lox*P system a very simple and powerful tool for mouse genomic engineering. 

Cre*-lox*P recombination in ES cells can be easily generated for deletions of small regions (<100 kb) with insertion of two *lox*P sites in a direct orientation and cis configuration [69, 70]. However, to obtain larger deletions or duplications with *lox*P sites on different chromosomes the frequency of the recombination is too low and requires to be selected through the restoration of a positive selection marker [71-75] or by the deletion of a negative selection marker [76-79]. To this end, targeting vectors were designed, containing the 5’or 3’ part of the *HPRT* minigene with *lox*P located downstream and upstream respectively [71]. Use of this technique has been largely facilitated by the creation and the allocation of specific targeting vectors [80] that are now mapped on the mouse genome (Mutagenic Insertion and Chromosome Engineering Resource [MICER], http://www.sanger.ac.uk/Post-Genomics/mousegenomics/) [81], avoiding the fastidious and time-consuming task of constructing the targeting vectors. In addition, retroviral vectors can be used to target the second *loxP* site providing new *loxP* sites nested insertions extending from a few kb to several Mb around defined loci [82]. 

Although Cre-*lox*P recombination is a very powerful and convenient technique, it has to be carefully planned as its efficiency depends on many factors. Obtaining deletion, duplication or inversion depends on the position (cis or trans) of the *lox*P site, the relative orientation of the two *lox*P sites and on the cell cycle stage during which the Cre-mediated recombination occurs [83, 84]. Cre-mediated recombination efficiency depends on the recombinase activity [85], the region targeted that could induced ES cell lethality after deletion [86], the genetic distance between the *loxP* sites in a cis configuration (10%-0.1%) and the configuration of the *lox*P sites [71, 84, 87]. The size of the deletions is limited by haploinsufficiency of genes within the deleted interval that can trigger ES cell lethality and can be circumvented by making balancer deletion/duplication using *trans* recombination. 

The possibility of manipulating large chromosomal regions has provided the scientific community with the opportunity to develop mouse models of CGS. Models developed for for del22q11.2 (DiGeorge syndrome) [88, 89] enabled to identify *TBX1* as a candidate gene for the pathology [90]. Modeling Down syndrome (DS), the only viable autosomal aneuploidy in human, requires multiple chromosomal rearrangements as the around 300 human chromosome 21 ortologs are found in three syntenic regions on mouse chromosomes (Mmu) 10, 17 and 16. The phenotypic analysis of the Ts65Dn and Ts1Cje models obtained in a random manner and trisomic for part of the MMU16 [91-93], the Ts1Rhr trisomic for a ~5 Mb Down syndrome critical region (DCR) [72, 94] and the Ts1Yah model trisomic for the syntenic region on Mmu17 [95], both generated by chromosomal engineering, start to reveal the complexity of the genetic interactions at the origin of the disease. Mouse models have also been created for the Smith-Magenis [96-99] and Prader Willi [100] CGS, and to study large genes or cluster of genes such as the *HoxD* genes cluster [70], the Duchenne muscular dystrophy gene [75], the amyloid precursor protein gene [76] or the *Nf1* gene [77]. *Cre/loxP* chromosome engineering in mouse ES cells was also developed to generate translocations or deletions similar to those found in cancer [86, 101-105]. Finally, in 2009, a mouse model was created for the first time to model a polygenic disease [106]. Autism, a common heterogeneous psychiatric disorder is a developmental brain disease characterized by deficit in social interaction and communication, and stereotyped repetitive behaviour. Autism has been shown to have a strong genetic basis with chromosomal anomalies accounting for 10 to 20% of the cases [107]. Among these anomalies, chromosome 15q11-13 duplication is the only recurrent cytogenetic aberration that could be associated with autism. Nakatani and collaborators (2009) generated a 6.3 Mb duplication of the conserved linkage group on Mmu7 and showed that the mouse model generated recapitulated aspects of human autism such as social abnormalities and increased anxiety and fear, providing a new tool to decipher the physiological and molecular mechanism underlying this pathology. 

## II. *IN VIVO* GERMLINE CRE-*LOX*P RECOMBINATION

**Fig. (1):**
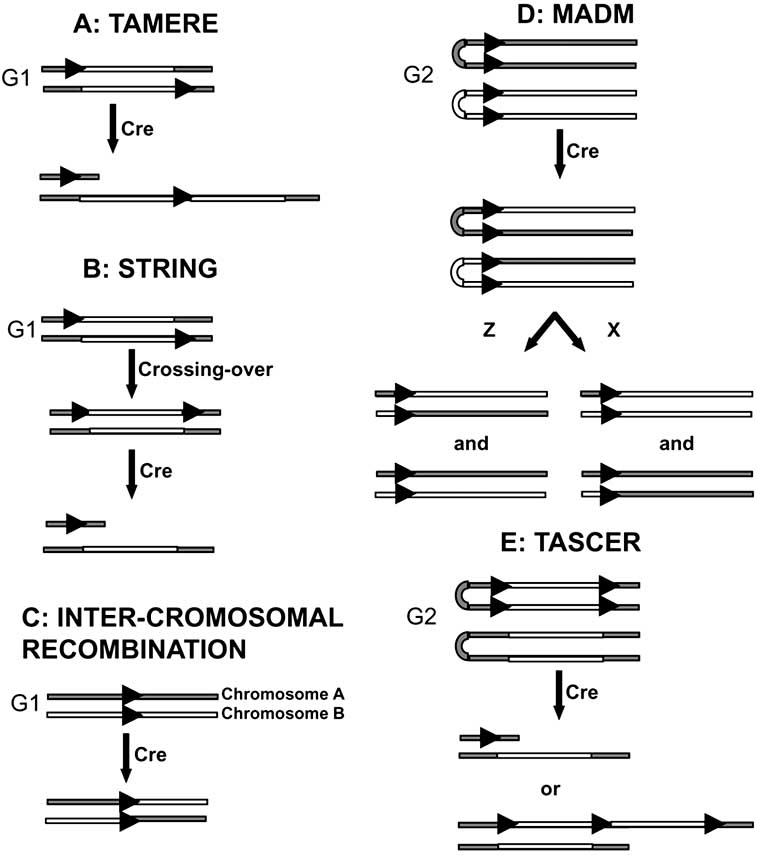
**Schematic representation of the different *in vivo* strategies developed to model CNV in the mouse.** (**A**) In the TAMERE strategy, *lox*P sites are inserted in a trans configuration and expression of the Cre recombinase during meiosis enables to generate cells containing a duplication with the reciprocal deletion. (**B**) The STRING approach takes advantage of the classical crossing-over to bring the two *lox*P sites into a *cis* configuration to generate a deletion. (**C**) Inter-chromosomal recombinations are obtained by introducing *lox*P sites at the site of the desired rearrangement and expressing the Cre recombinase. (**D**) The MADM strategy base on Cre-mediated inter-chromosomal recombination and G2-X segregation during mitosis, offers the possibility to introduce mosaicism *in vivo*. (**E**) The TASCER strategy takes advantages of the G2 stage of the S phase to recombine *lox*P sites in *cis*, leading to the deletion and the duplication of the sequence surrounded with the *lox*P.

Creating chromosomal rearrangements in the whole organism offers an alternative to the *in vitro* protocol that is very labor-intensive and requires many rounds of ES cells genomic manipulations (Fig. **1**). It requires that the *lox*P sites recombination occurs either in germ line cells during meiosis or ubiquitously in all of the tissues or in early embryogenesis. The rearrangements can then be transmitted to the progeny. This can be achieved by using Cre transgenic lines such as Sycp1-Cre [108], *Zp3-Cre *[109, 110], Protamine-Cre [111], *CMV-Cre *[112], R26Cre [113] or *Hprt-Cre *[114]. Most of the large *in vivo* rearrangements are deletions that are obtained from two *lox*P sites in the same relative orientation inserted in cis in ES cells [70, 115-117]. The frequency of recombination in cis decreases with the increasing distance between the *lox*P sites (0.3%-1%), but still remains high enough to be workable up to distances reaching 28 Mb [118]. For *lox*P sites that are originating from different mouse founder lines and hence are not in cis, deletion can still be obtained by mating the two founder lines and selecting for recombination after classical crossing-over, a technique called STRING for Sequential Targeted Recombination INduced Genomic rearrangement [118]. Similarly, inversions have been obtained *in vivo* with two *loxP* sites located in the same chromosome but with a reverse orientation [115, 119]. The different ubiquitously expressed Cre transgenic lines are not all similarly efficient in making the recombination and can lead to mosaic individuals in the first generation [73, 120, 121]. An alternative to the ubiquitous *Cre* transgenic lines is the *Zp3-Cre *in which *cre* expression is controlled by regulatory sequences from the mouse zona pellucida gene, expressing exclusively in the growing oocyte prior to the completion of the first meiotic division. But this transgenic *Cre* line was also shown to produce some mosaicism in a mouse model for DiGeorge del22q1 syndrome [122].

Creating chromosomal duplications requires that the two *lox*P sites are on homologous chromosomes (*trans*) and is only possible when the two homologous chromosomes becomes physically close together. A strategy called TAMERE for TArgeting MEiotic Recombination was designed by Herault *et al*. (1998) [108] that makes use of a Cre under the control of the Synaptonemal Complex 1 promoter that is expressed during prophase of meiosis in male spermatocytes when chromatid pairs are closely aligned [123], enabling trans recombination [115, 124, 125]. However, for larger genomic rearrangements, while *in vivo* recombination is still effective for cis configurations up to 28 Mb [118], no rearrangement was obtained for a distance of 3.9 Mb [126] and hence seems to be limited to short-distance recombination. The TAMERE strategy has been used extensively to rearrange and study the *HoxD* complex region [108, 119, 127, 128] and has also been used to address the functional relationship between *PrPc* and *Dp*l, two genes involved in Prion disease, by making a double deletion of these two genes that are separated by 16 kb [125]. 

Beside the Cre-*lox*P system described here, the Flp-*FRT* system is another tyrosine site-specific recombinase (SSR) system that has been used as tool for DNA and genome engineering. However, FLP recombinase was found to be less efficient than Cre, partly due to an unsuitable optimum temperature for mammalian cells [129]. Recently, a new Cre-like recombinase, called Dre, was identified from P1-related phage D6 by Sauer and McDermott (2004) [130]. Dre recombinase has DNA specificity for a 32 bp DNA site (rox) distinct from the *lox*P and shows no cross-recombination with the Cre-*lox*P system. Like the Cre, it catalyzes both integrative and excisive recombination and requires no co-factor. The Dre-*rox* system was validated in the mouse by Anastassiadis and collaborators (2009) [131] who tested the efficiency of Dre-rox recombination *in vivo* by developing ubiquitous mouse lines based on the CAGGs promoter [132] and the ROSA26 locus. This new SSR provides a new useful toolkit for mouse genome engineering. Finally, recent applications for large serine SRRs such as phiC31 and phiBT1 [133] in eukaryotic genome engineering have emerged as new additional tools.

Association of the Cre-*lox*P system with transposon-based approaches should considerably speed up the generation of large-scale rearrangements *in vivo*. Transposons are mobile genetic elements that function as a bipartite system with a transposase to move around to different positions within the genome, potentially perturbating gene function. They have been extensively used for random mutagenesis and have been shown to be efficient both *in vitro* in ES cells and *in vivo* in the germline or somatically [134-141]. The most widely studied transposon/transposase system for mammalian mutagenesis is the Sleeping Beauty transposon of the Tc1-like mariner family [142]. Transposons have the advantage that they are active *in vivo* and only require breeding of transgenic mice, their insertion sites can be defined thanks to the transposon serving as a tag. More recently, an alternative transposon called Piggybac, derived from the cabbage looper moth (*trichoplusia ni*) has been developed, that can carry larger payloads between its transposon repeats [138]. PiggyBac transposons offer the possibility to easily distribute *lox*P sites throughout the mouse genome and, associated with the Cre-*lox*P technology, permit to generate rapidly and easily genome-wide chromosome rearrangements [121]. 

## III. SOMATIC GENETIC ENGINEERING

Mosaicism in copy number variation has been observed in human [143]. It is a direct consequence of the missegregation of a chromosome during mitosis: while the two copies of one homologous chromosome migrate in one daughter cell that will be trisomic, the second will have only one copy being now monosomic.

The percentage of aneuploid mosaicism depends on the developmental stage, the lineage of the cell that is affected and its viability, and the type of chromosomal rearrangement produced. If the rearrangement occurs early during embryogenesis, the mosaicism can be generalized to all cell lineages and can result in major phenotypic changes that are often lethal. For example, in Turner syndrome more than 98% of conceptuses that are 45, X do not survive to term and only mosaic are thought to be compatible with life [144]. 

Mosaic aneuploidy can be registered in all somatic cell populations contributing to normal inter individual variability, with a frequency of 1.25–1.45% per chromosome and a percentage of aneuploid cells above 30% reported in blood cells [145] and brain [146-148]. Reasons for the presence of such aneuploid cells and its consequence remain for the moment speculative. 

Consequences of somatic aneuploid mosaicism can be analysed in the mouse by generating segmental aneuploid mosaicism in mouse tissues using the Cre-*lox*P system during the G2 phase of mitosis. Two different strategies were developed using this system. Zong *et al*. (2005) [149] developed a technique utilizing Cre-mediated inter-chromosomal recombination prior to cell division (mitotic recombination) and took advantage of the G2-X segregation during mitosis to induce recombination events between homologous chromosomes in somatic cells and to mark the resulting daughter cells with different genotypes. They called this technique Mosaic Analysis with Double Markers (MADM). In addition to create and study sporadic loss of heterozygosity, this technique can also be used with a pre-introduced deletion or duplication on one chromosome to create CNV mosaics composed of wild-type daughter cells bearing the normal allele or mutant cells containing the allele with the deletion or the duplication. Another strategy takes advantages of the property of the Cre recombinase to react on G2 phase of mitosis to directly generate different types of cells with either the duplication or the deletion of one chromosomal region targeted with the *lox*P sites [150]. The TASCER (Targeted Asymmetric Sister Chromatid Recombination) takes advantage of the presence of *lox*P sites on sister chromatids after replication of DNA in G2 phase during mitosis in order to generate *in vivo* cells harbouring microdeletions and microduplications. *Lox*P sites were inserted on the same chromosome in a cis configuration separated by up to 2.2 Mb. Upon mating Cis/+ mice with a general Cre deleter line, mosaic animals were obtained with cells containing either the deletion or the duplication. Indeed, if *lox*P recombination occurs after DNA replication, in addition to the expected deletion, the corresponding duplication was also obtained corresponding to the intermediate reaction product containing three *lox*P sites. The duplication with presence of three *lox*P sites represents the intermediate product between the double cis configuration with four *lox*P sites of the G2 stage cells and the deletion end product. This is the first report of an *in vivo* generated duplication coming from a cis configuration, presumably because, in most reports, *lox*P sites were not as distant, resulting in a better efficiency avoiding the presence of the intermediate genomic duplication configuration. Hence, the increased distance tends to decrease the Cre-mediated recombination frequency [87] somehow preserving the chromosome with a large duplication. Using TASCER on two chromosomal regions homologous to the telomeric part of human chromosome 21, cells with partial monosomy and trisomy were efficiently recovered at a relatively constant level in numerous different tissues. The TASCER mechanism necessitates that the Cre is particularly active during the G2 phase during cell proliferation. This phenomenon has been described *in vitro* in embryonic stem cells [84, 151] but with much less efficiency than *in vivo*. The reason for this discrepancy might be that expression of the Cre is more stable *in vivo* and that the *in vivo* environment is more favourable to the viability of the aneuploid cells. Change in the chromatin structure, with complexes such as the cohexin one inducing physical connection of sister chromatids, can also facilitate the mitotic recombination [152, 153].

Efficiency of the TASCER is also depending of the type of Cre transgenic line used, as these do not express the Cre recombinase with the same efficacy. Hence, Duchon *et al*. (2008) [150] were able to induce up to 2.2 Mb deletions and duplications with a set of three different Cre lines, but could not obtain a larger modification of 9.2 Mb that might require a Cre line with a stronger promoter such as the CAG promoter inserted in the *Hprt *locus of the *Hprt1^tm1(Cre)Mnn^* mouse line, which has been used successfully in a recent study [114]. The fact that cells bearing the duplication was found in many different organs despite the less stable presence of three *lox*P sites [154] indicates that the trisomic state might be beneficial in some tissues and point out at the necessity to be very careful when using the Cre-*lox*P system *in vivo* to generate deletions. 

Whereas chromosomal mosaicism has been observed in pathological conditions, such as Turner syndrome [155] or in cancer [156], the TASCER publication did not report any dramatic changes in the survival of trisomic and monosomic cells produced. In some tissues such as the muscle, this can be explained by compensatory effects due to their organization in a plurinuclear syncitia. However, in other non syncitial tissues, other mechanisms such as non-cell autonomous effect should be proposed to explain this phenomenon. 

Although mosaic aneuploidy and CNVs have been reported in nonpathological conditions in different cell populations such as neurons [51, 52, 146-148, 157-159], their role and biological consequence to the organ structure and function in health and disease remains under explored. In the brain, mosaic DNA rearrangements have been suggested to contribute to the mechanism for neuronal cell diversification [160]. However, these are only suppositions and there is to date little evidence supporting a functional consequence for mosaic CNV in nonpathological conditions. To answer those questions, future studies need to examine the effect of somatic CNV on cell physiology. TASCER that results into substantial segmental aneuploid mosaicism, offers the possibility to explore consequences of such a phenomenon in a model organism. It further can be used to to induce partial aneuploid conditions such as those detected in cancer cells and to study further the consequence of loss or gain of copy number for chromosomal regions during tumorigenesis [156], avoiding the fastidious need of gene-by-gene inactivation or ES-cell MICER recombination system to study such phenomenon. TASCER hence offers new perspective for the generation and analysis of CNV variation in health and disease.
